# The Influence of Reaction Time on Non-Covalent Functionalisation of P3HT/MWCNT Nanocomposites

**DOI:** 10.3390/polym13121916

**Published:** 2021-06-09

**Authors:** N.M. Nurazzi, N. Abdullah, S.Z.N. Demon, N.A. Halim, I.S. Mohamad

**Affiliations:** 1 Centre for Defence Foundation Studies, National Defence University of Malaysia, Kem Sungai Besi, Kuala Lumpur 57000, Malaysia; mohd.nurazzi@gmail.com (N.M.N.); zulaikha@upnm.edu.my (S.Z.N.D.); norhana@upnm.edu.my (N.H.A.); 2 Department of Diploma Studies, Faculty of Mechanical Engineering, University Teknikal Malaysia Melaka, Hang Tuah Jaya, Durian Tunggal, Melaka 76100, Malaysia; imran@utem.edu.my

**Keywords:** CNT, MWCNT, non-covalent functionalisation, polythiophene, P3HT, reaction time

## Abstract

Non-covalent functionalisation of the carbon nanotube (CNT) sidewall through polymer wrapping is the key strategy for improving well-dispersed CNTs without persistent alteration of their electronic properties. In this work, the effect of reaction time on regioregular poly (3-hexylthiophene-2,5-diyl) (P3HT)-wrapped hydroxylated multi-walled CNT (MWCNT-OH) nanocomposites was investigated. Five different reaction times (24, 48, 72, 96, and 120 h) were conducted at room temperature in order to clearly determine the factors that influenced the quality of wrapped MWCNT-OH. Morphological analysis using Field Emission Scanning Electron Microscopic (FESEM) and High-Resolution Transmission Electron Microscope (HRTEM) analysis showed that P3HT successfully wrapped the MWCNT-OH sidewall, evidenced by the changes in the mean diameter size of the nanocomposites. Results obtained from Raman spectroscopy, X-ray Photoelectron Spectroscopy (XPS) as well as Thermogravimetric Analysis (TGA) showed a significant effect of the wrapped polymer on the CNT sidewall as the reaction time increased. Overall, the method used during the preparation of P3HT-wrapped MWCNT-OH and the presented results significantly provided a bottom-up approach to determine the effect of different reaction times on polymer wrapping to further expand this material for novel applications, especially chemical sensors.

## 1. Introduction

Since the discovery of polyacetylene in 1977 by Hideki Shirakawa et al., research in the field of conductive polymers has heavily impacted the discovery and development of π-conjugated conductive polymers [1]. Since then, many studies have been conducted by various researchers from different fields using leading conductive polymers, such as polypyrrole (PPy), polyaniline (PANI), polythiophene (PTh), poly (3,4-ethylenedioxythiophene) (PEDOT), and poly (p-phenylene vinylene) (PPV). These conductive polymers demonstrated great potential for integration into future optical and electronic devices due to their capacity to transition between semiconducting and conducting states, as well as the ability to alter mechanical properties by controlled doping, chemical modification, and stacking or creating composites with other materials [2]. In general, conductive polymers possess alternating single (σ) and double (π) bonds, and these π-conjugated systems provide the conductive polymers with intrinsic optical, electrochemical, and electrical and electronic properties [3]. However, the development of the properties of conductive polymers has not been completely proportionate with those of their metallic and inorganic semiconductor counterparts. Therefore, conductive polymers have been modified or hybridised with other heterogeneous material or other carbonaceous material components to overcome and improve their inherent boundaries in terms of solubility, conductivity, and long-term stability [4]. Amongst the conductive polymers and the π-conjugated polymers, PTh and its derivatives have shown promising characteristics, which were comparable to those of both PANI and PPy, for wide applications such as in chemical and biosensors [5,6,7,8], organic photovoltaics (OPVs) [9,10,11], electronic magnetic shielding (EMI) [12,13], battery [14,15], microwave absorption [16,17], water purification devices [18,19], and hydrogen storage [20,21].

PTh-based nanocomposites containing nanocarbon species, such as graphene, carbon nanofibers (CNFs), and CNTs, were developed and show promising results in the targeted applications [22,23,24,25]. These nanocarbon species improved the structural ordering of the nanocomposite chains and facilitated delocalisation of the charge carriers, resulting in enhanced conductivity. Some conductive polymers can behave like semiconductors due to their heterocyclic compounds, which display physicochemical characteristics such as solubility, hydrogen bonding, surface activity, thermal expansion, and electrical conductivity. As a result, reversible changes in the sensing layer’s conductivity could be detected upon polar chemical adsorption on the sidewall at room temperature [26]. This effect is believed to be caused by the charge transfer between gas molecules and the polymer or swelling of the polymer film’s [27]. Amongst the carbonaceous materials considered to date, CNT has been widely investigated because of its excellent electrical, mechanical, and thermal properties [28]. Since then, various techniques to incorporate CNT in polymer matrices were designed with a desire to fabricate new advanced materials with multifunctional properties. The exceptional mechanical properties associated with CNT vary in the literature regarding the exact properties of CNT. Theoretical and experimental results have shown an extraordinarily high elastic modulus, greater than 1 TPa (the elastic modulus of diamond is 1.2 TPa) and reported strengths 10 to 100 times higher than those of the strongest steel at a fraction of the weight [29]. CNT also has superior thermal and electric properties: thermally stable up to 2800 °C in a vacuum, electrical conductivity is about 10^3^ S/cm (the electric current-carrying capacity is 1000 times higher than those of metals like copper), thermal conductivity is about 1,900 W m^−1^ K^−1^, (the thermal conductivity is about twice as high as that of diamond [30,31,32]), and the current density of individual metallic single-walled carbon nanotubes (SWCNT) is about 4 × 10^9^ A/cm^2^, which is 1000 times higher than that of copper wires [33].

Besides the exceptional characteristics mentioned above, low compatibility and limitation of dispersion of CNTs in polymeric matrices occurred, but the interaction between CNTs and the polymer remained weak. CNTs were in the form of long bundles due to stabilisation by π–π electron interactions and high surface energy. CNTs incorporated into the polymeric matrices was an attractive method to combine and complement the optical, electrical, and mechanical properties of individual CNTs with the unique properties of the desired polymer. These unique properties make CNTs an ideal reinforcing agent in a number of applications [34]. In previous research on homogeneously dispersed CNTs as a conductive filler in polymer matrices, researchers covalently functionalised the CNT sidewall with a monomer such as thiophene [35,36] and specific functional groups or active elements such as carboxylation, amides, etc. [37,38,39,40,41,42,43]. Based on the aforementioned studies, the covalent modification towards CNT composites produced high stability functionalisation. In particular, this mechanism would lead to the efficient load transfer from the polymer/CNT matrix through the covalent bonding [44]. However, in most cases, covalent functionalisation changes the structure of the CNTs, thereby degrading their unique electrical and mechanical properties. Furthermore, functionalisation causes shortening of the CNTs, which reduces the advantage of CNTs in regard to the ratio aspect [29].

The optimisation strategy for the improvement of CNT dispersion in polymers matrices was done by Liu and Choi (2012). The dispersion is a spatial property, whereby the individual CNTs are spread with a roughly uniform number density throughout the continuous polymer matrix. The challenge is to separate the nanotubes from their initial aggregated nature, which is usually achieved by local shear forces. Direct manual mixing of CNTs with polymer resin, though the simplest approach, did not create sufficient local shear force and therefore led to poor dispersion of CNTs inside the polymer matrix. A more effective separation and dispersion of CNT bundles requires the overcoming of the inter-tube van der Waals attraction forces. Physical approaches such as shear mixing, mechanical stirring, further sonication, ball milling, and micro-bead milling processes have been employed for this purpose. Although these techniques might appear very different, they are all governed by the transfer of physical shear stress onto nanotubes, which break down the bundles. Usually, dispersion via shear mixing is only achievable for specific types of CNTs, with a high shear rate in a rather viscous medium. Huang et al. (2006) demonstrated that nanocomposites containing high loading concentrations of CNTs (up to 7 wt.%) could be dispersed via this technique [45]. However, the processing time significantly increases as the loading concentration rises. More importantly, shear mixing tends to sectionalise CNTs into shorter length, thereby reducing their conductivity significantly, an undesired attribute for nanocomposite, which was intended for use as a sensor material. 

Another concern of the optimisation is the sonication frequency. A higher power of sonication, greater than 500 W, is desired as it provides a higher shearing force to break down the CNT bundles. Nevertheless, prolonged exposure could possibly damage or shorten the nanotubes that finally leads to decreased conductivity of the CNTs. For the dispersion related to optimisation of the mechanical stirring, a specific speed (rpm) could shorten the dissolution time. Prolonging the additional time of mechanical stirring led the dispersion to become less stable, and CNT agglomerations were visibly seen. Therefore, the use of non-covalent functionalisation, which maintained the CNT structure, was proposed as a key strategy to achieve well-dispersed CNTs in PTh matrices and better PTh wrapping towards the CNT sidewall. Referring to Bose et al. (2010), non-covalent functionalisation was considered an efficient alternative strategy to tailor the CNT and polymer interface and preserve the reliability of the nanotubes [46]. This route is particularly attractive because of the possibility of adsorbing various groups of ordered architectures on the CNT sidewall without disturbing the extended p-conjugation of the nanotubes. Besides the effect of functionalisation during the preparation of polymer wrapping towards the CNT sidewall, the effect of reaction time is also one of the crucial criteria. Hence, in this paper, a composition of 1:1 of P3HT and MWCNT-OH was used, and the physical properties of nanocomposites under the different reaction times were investigated. FESEM and HRTEM were performed to study the structural and the wrapping dispersion of nanotubes in a polymer matrix, whereas, the structural, morphological, and thermal stability properties were evaluated by Raman, XPS, and TGA, respectively.

## 2. Experimental Section

### 2.1. Materials

Commercially available MWCNT-OH (purity, >95%) was purchased from Nanostructured & Amorphous Materials, Inc., Houstan, Texas, USA. Regioregular P3HT (molecular weight, Mw 50,000 to 100,000; purity, ≥90%) and organic solvent tetrahydrofuran (THF) were purchased from Sigma–Aldrich, Selangor, Malaysia. Methanol (CH_3_OH), a solvent used for washing, was supplied by R&M Chemicals, Selangor, Malaysia. The materials and solvent were used as received without further purification.

### 2.2. Preparation of P3HT/MWCNT-OH Nanocomposites

**Figure 1:**
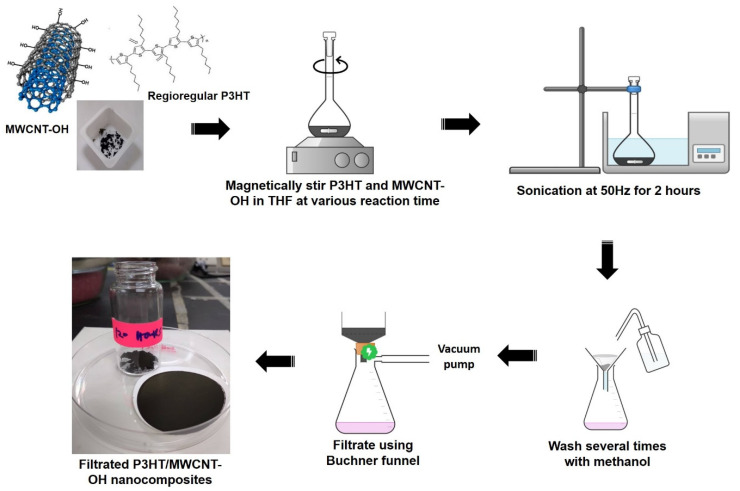
Schematic procedure for preparation of P3HT-wrapped MWCNT-OH nanocomposites.

The preparation of P3HT/MWCNT nanocomposites was adopted from a previous methodology [47], with a revised version. The schematic procedure followed for preparation of the nanocomposites is presented in Figure 1. Approximately 5 mg of MWCNT and 5 mg of P3HT were added to a 25 mL volumetric flask consisting of a magnetic bar (0.5 (L) cm × 0.2 (D) cm). Then, 5 mL of THF was poured into the volumetric flask. The volumetric flask was placed in a ceramic heating plate, and the suspension was stirred consistently at a speed of 650 rpm for 24 h at 50 °C (sample labelled as MWCNT-24). The reaction mixture was then immersed in a water bath and sonicated at a frequency of 50 Hz for 2 h at room temperature. The resultant precipitate of the P3HT/MWCNT nanocomposite was then carefully washed several times with methanol and further filtered using a vacuum Buchner funnel. The obtained black powder was dried at 25 °C for 24 h (MWCN-24). With the same amount of materials and procedure, the preparation of nanocomposites was repeated at different reaction times for 48, 72, 96, and 120 h and labelled as MWCNT-48, MWCNT-72, MWCNT-96, and MWCNT-120, respectively. The yield of the nanocomposite from each set of experiments was around 80 to 90%.

### 2.3. Characterisation Methods

The structural analysis through the Raman spectroscopic measurement was carried out using Renishaw inVia Reflex Confocal Micro Raman System (Renishaw plc, Wotton-under-Edge, UK). The HPNIR laser for sample excitation was set at a wavelength of 787 nm, 1200 mm^−1^ gratings, and the magnification was a 100× objective lens. The chemical state of the element compositions of P3HT/MWCNT was analysed using an auger electron spectroscope with an x-ray photoelectron spectrometer (XPS), Kratos/Shimadzu (Shimadzu Corporation, Kyoto, Japan). The binding energy values of XPS lines were calibrated using Al Kα radiation at 1000 eV (hv = 1000 eV) and a spot size of 100 µm. The surface morphological analysis and the diameter size distribution of the nanocomposites were characterised using a Field Emission Scanning Electron Microscopic (FESEM), Carl Zeiss Gemini FESEM 500 (Carl Zeiss AG, Jena, Germany). Prior to the analysis, samples were initially coated with gold for about 1 min in order to avoid charging. The effectiveness of polymer wrapping onto MWCNT was also overserved under a High-Resolution Transmission Electron Microscope (HRTEM), JEOL JEM 2,100F HRTEM (Tokyo, Japan) at an acceleration voltage of 200 kV. For HRTEM analysis, the sample was initially dispersed in acetone by ultra-sonication for 60 s. Thereafter, a drop of the suspension was transferred onto a carbon-coated copper grid and mounted on the microscope, and the images were recorded. The TGA was conducted using a TGA-DSC HT 3 analyser (Mettler Toledo, Selangor, Malaysia) from a temperature of 25 to 900 °C at a heating rate of 10 °C/minute under a nitrogen atmosphere to study the stability of the prepared nanocomposites.

## 3. Results and Discussion

### 3.1. Raman Spectroscopy Analysis

**Figure 2:**
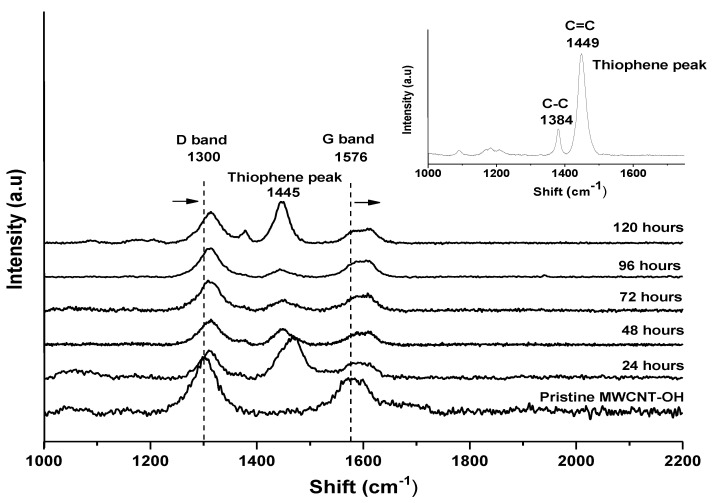
Raman spectra of (**a**) pristine P3HT, (**b**) pristine MWCNT-OH and P3HT/MWCNT-OH nanocomposites prepared at different reaction times.

Raman spectroscopy was used to study the possible interactions between pristine MWCNT-OH and the P3HT. The pristine MWCNT-OH spectrum was compared with the P3HT-wrapped MWCNT-OH nanocomposites that were prepared at different reaction times, as can be seen in Figure 2. The summarised intensity ratio between the D band and G band is presented in Table 1. From the Raman spectra it could be observed that pristine P3HT had two distinct peaks at wavelengths of 1384 cm^−1^ and 1449 cm^−1^, which corresponded to the C-C skeletal stretching vibrations and C=C skeletal stretching of the thiophene ring deformation of the side chain of P3HT, respectively. The Raman spectra of MWCNT-OH demonstrated two prominent peaks characteristic at 1300 cm^−1^ (D band) attributed to the disorder and imperfection of the carbon crystallites and sp^3^ vibration present in the MWCNT-OH structures [48]. Peaks at 1571 cm^−1^ (G band) corresponded to one of the two E_2g_ modes corresponding to stretching vibrations in the basal plane (sp^2^ domains) of single-crystal graphene [49]. After the non-covalent functionalisation of MWCNT-OH with P3HT, both the D band and G band were shifted to a higher wavelength and an additional peak was observed at 1445 cm^−1^, which represented the characteristic of protonated P3HT. A relative increase and shift in the D band led to the increased intensity ratio, which indicated the slightly defected structures of the MWCNT-OH embedded by the P3HT [6,50].

The intensity ratio data presented in Table 1 showed that increasing the reaction time of the suspension from 24 h to 120 h resulted in a linear increase in the intensity ratio of I_D_/I_G_ Raman spectra from 0.83 (pristine MWCNT-OH) to 0.98 (MWCNT-120). This trend indicated that pristine MWCNT displayed a slightly less disordered structure compared to nanocomposite samples. Gomez et al. (2016) increased the intensity ratio of the Raman spectra corresponding to a higher number of sp^3^ carbon, which was generally attributed to the presence of more structural defects [51]. The more structural defects in this study referred to the successful functionalisation of the P3HT towards the wall of the MWCNT-OH as a non-covalent interaction. This interaction led to less penetration of the electron for the detection of the graphitic surface (G band) rather than the penetration of the electron on the wrapped area classified as a defective and imperfect surface.

Additionally, since the glass transition temperature (T_g_) of P3HT was low at 12 °C [52], the longer contact time between the nanotubes and P3HT in suspension was expected to increase the heat generation upon the stirring process over time. This synergised the wrapping process of the P3HT onto the MWCNT wall and tips, whereas the additional new peak at 1449 cm^−1^ for all the nanocomposite samples was due to C=C stretching vibrations from polymer P3HT. The intensity of this peak was prominent for sample MWCNT-24 and showed a decreasing of trend as the reaction time increased. This indicated the functionalisation of P3HT towards the MWCNTs-OH sidewall was possibly well formed towards the success of P3HT wrapping (Figure 2). However, for sample MWCNT-120, a well-defined peak could be seen at 1445 cm^−1^, which indicated that more a concentrated thiophene group was present on the nanotube wall. Furthermore, interactions between MWCNT-OH and P3HT blended for nanocomposites at 24-hour reaction time caused a shift in the D band of about 14 cm^−1^, from 1297 to 1312 cm^−1^, and in the G band of about 80 cm^−1^ from 1571 to 1492 cm^−1^. For the longer reaction time at 120 h, the nanocomposites caused a shift in the D band of about 8 cm^−1^, from 1297 to 1398 cm^−1^, and in the G band of about 26 cm^−1^ from 1571 to 1427 cm^−1^. The shifting of both the D band and G band in the Raman spectra was due to the wrapped polymer on the nanotube walls and this was in line with the observation from FESEM images and was supported by the diameter size distribution histogram. A clear enhancement in the intensity ratio observed on P3HT wrapping was also reported by other researchers, which correlated with the effect of non-covalent polymer wrapping on the CNT sidewall [53,54,55,56].

### 3.2. XPS Analysis

**Figure 3:**
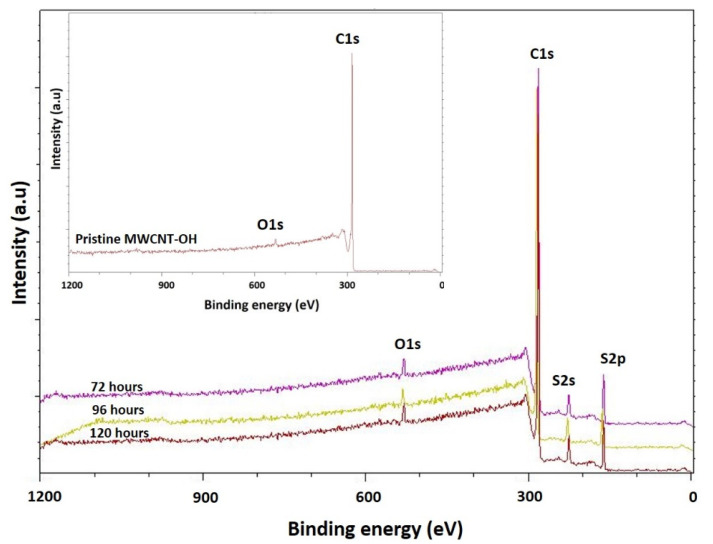
XPS spectra of pristine MWCNT-OH and P3HT/MWCNT-OH nanocomposites prepared at different reaction times.

The presence of the functional group on the sidewall of P3HT/MWCNT-OH nanocomposites, prepared at different reaction times, was further investigated using XPS analysis (Figure 3 and Table 2). It could be seen that the intensity peak for pristine MWCNT-OH exhibited an intense peak at 283.45 corresponding to C 1s (97.25%) and a relatively intense peak at 531.6 eV corresponding to O 1s (2.75%) [57]. There was no S 2s or S 2p spectrum in the case of the MWCNT-OH. The S 2p core-level spectrum of P3HT and P3HT/MWCNT-OH could be deconvoluted into at least two spin-orbit-split doublet (S-2p_3/2_ and S-2p_1/2_) peaks at approximately 165.1 eV and 165.8 eV, respectively, which were attributed to the neutral sulfur atoms [58]. In a study by Karim et al. (2006), the successful wrapping of PTh towards the CNT sidewall could be observed by the two peaks of S 2p, which appeared at 163.7 and around 230 eV for S 2s [50]. This means that the chemical environment of the S element, in pure P3HT and P3HT/MWCNT-OH, was almost identical. The MWCNT-OH nanocomposites for all samples wrapped with P3HT showed a decrease in carbon and oxygen contents, whilst the peak for the sulfur content started to appear for all nanocomposites samples. An enhancement in the intensity for all nanocomposite samples, such as P3HT/MWCNT-OH at 72 h, exhibited characteristic peaks of sulfur at 227 eV (S 2s) and 163 eV (S 2p) in addition to C 1s and O 1s peaks, which confirmed the presence of thiophene moieties from P3HT on the MWCNT-OH sidewall [59,60,61,62].

The P3HT/MWCNT-OH with 120 h of reaction time contained the higher atomic composition of P3HT at S 2s and S 2p, which was evident for the higher P3HT wrapped on the MWCNT-OH sidewall and increased the diameter size distribution (Table 3). The intensity of the carbon atomic percentage was found to decrease as the reaction time increased up to 120 h of reaction time, compared with the pristine MWCNT-OH carbon atomic percentage because the graphite surface of the MWCNT-OH was covered by the P3HT. This complemented the Raman results (Table 1), whereby prolonging the reaction time led to an increase in the defective band (D band), which was related to the Raman intensity ratio. The increased defective band indicated the graphitic or the carbon atoms had been covered or wrapped with the P3HT on the MWCNT-OH sidewall.

### 3.3. FESEM and Mean Diameter Size Distribution Analysis

**Figure 4:**
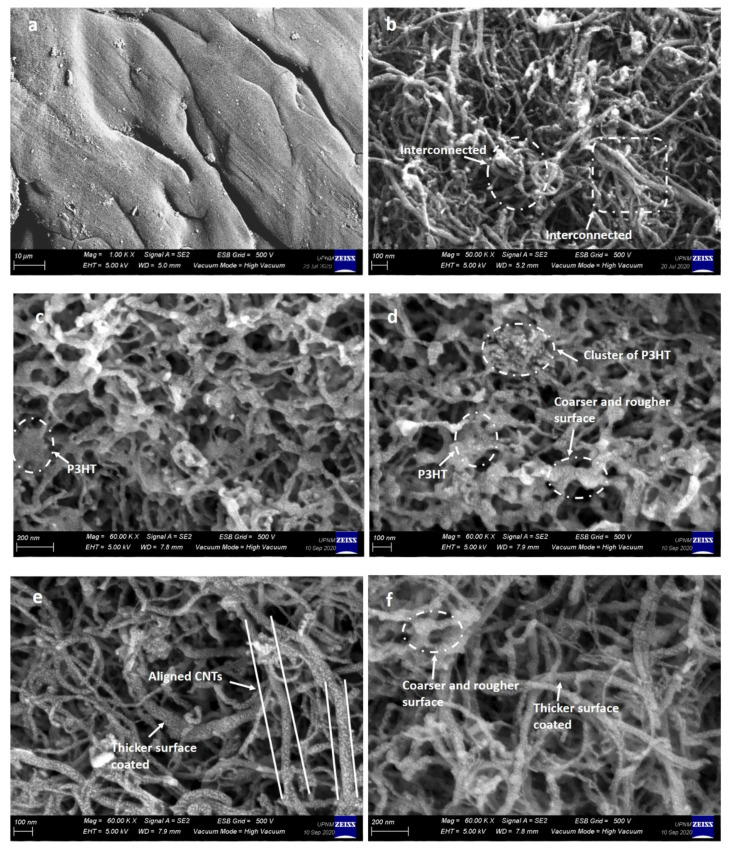

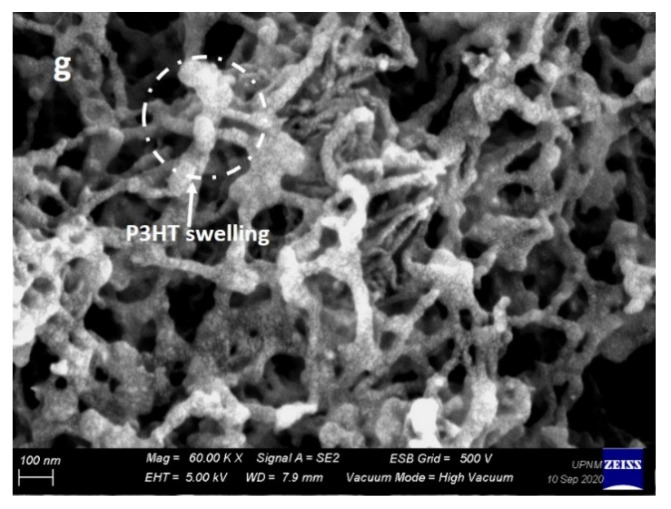
FESEM image of (**a**) pristine P3HT, (**b**) pristine MWCNT-OH, P3HT/MWCNT-OH nanocomposites at different reaction times (**c**) 24 h, (**d**) 48 h, (**e**) 72 h, (**f**) 96 h, and (**g**) 120 h.

Figure 4 depicts the texture surface morphology of pristine P3HT, pristine MWCNT-OH, and the nanocomposite images that were prepared at different reaction times under FESEM. As shown in Figure 4a, the pristine P3HT showed a smooth surface texture without any notable morphology, and it was clearly visible, whereas, the morphology of pristine MWCNT-OH in Figure 4b showed that the nanotubes were not well aligned and were randomly entangled. In certain areas, the nanotubes were highly interconnected with each other, and agglomeration of MWCNT-OH was highly visible. This behaviour was explained by the fact that due to strong intrinsic van der Waals forces, CNTs tend to hold together as bundles, which eventually leads to low solubility in many solvents and therefore poor dispersion when mixed into various polymers.

For the P3HT/MWCNT-OH nanocomposites, the surface texture became coarser than the pristine MWCNT-OH, indicating that they were successfully wrapped and covered by the P3HT matrix. From the optimised reaction time for polymer wrapping, the non-covalent π–π interaction and CH-π interaction took place on wrapping of P3HT over MWCNT-OH walls. From the images in Figure 4c,d for MWCNT-24 and MWCNT-48, it was observed that there was not much difference in the images. The FESEM image showed P3HT were agglomerates and were inhomogeneously distributed on the nanotube surface. Furthermore, highly saturated, thicker and unsmooth wrapping spots were observed in certain areas. The entangled nanotubes were still visible in certain areas. This might be due to insufficient contact time for the P3HT to overcome the van der Walls forces towards the nanotube surface that led to better dispersion and wrapping. For MWCNT-72 and MWCNT-96 nanocomposite images in Figure 4e,f, less tangling and better alignment of MWCNT-OH were observed. This was due to the successful covering and wrapping of the P3HT, which made the nanotube sidewall thicker. In some areas, a highly uniform coating of P3HT along the nanotube surface could be seen. However, bigger clusters and a coarser surface were clearly shown in the images observed for MWCNT-120 in Figure 4g. This might be attributed to the swelling of P3HT due to a longer reaction time that made it saturated in a certain area along the nanotube wall.

**Figure 5:**
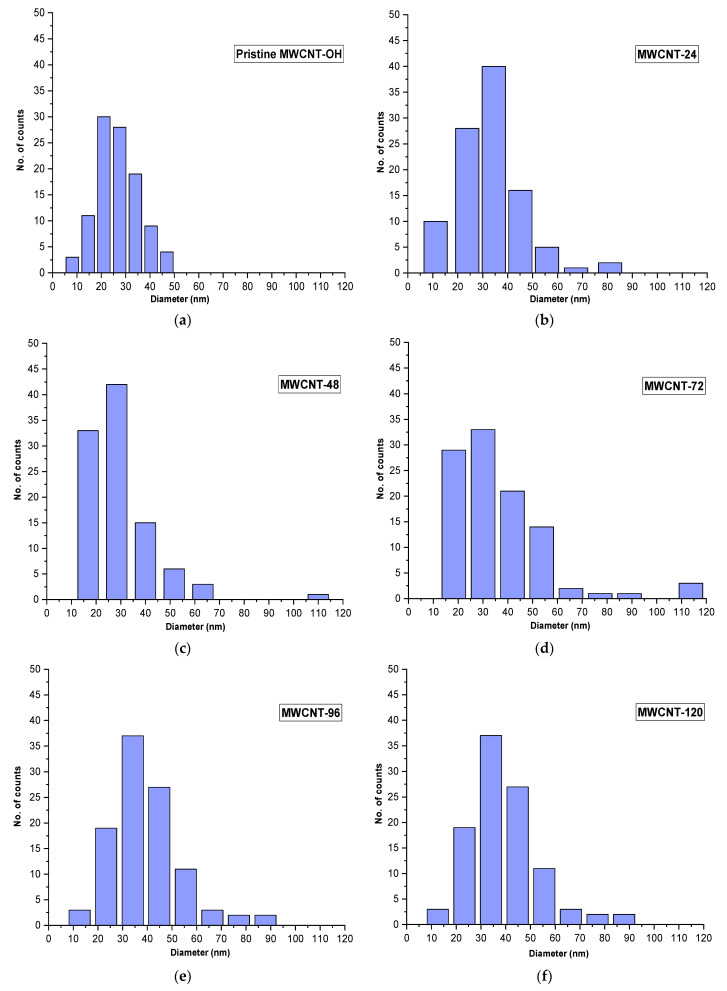
Histogram of diameter size distribution of (**a**) pristine MWCNT-OH and (**b**–**f**) P3HT/MWCNT-OH nanocomposites at different reaction times.

The mean diameter size of nanocomposites was obtained using ImageJ software based on 100 points plotted in FESEM images. The histogram average of the variation of the diameter size distribution and standard deviation data observed for pristine MWCNT-OH and the P3HT/MWCNT-OH is shown in Figure 5 and Table 3, respectively. An average diameter size (together with standard deviation (stdev)) of the nanocomposite continued to increase from 27 nm (stdev = 8.10), 33 nm (stdev = 12.48), 35 nm (stdev = 14.80), 42 nm (stdev = 18.30), 45 nm (stdev = 12.58), and 50 nm (stdev = 19.24). This showed that the increase in the reaction time of the suspension increased the effectiveness of P3HT wrapping towards the MWCNT-OH sidewall by the increased diameter of MWCNT-OH. This also correlated with the intensity ratio of I_D_/I_G_ Raman spectra, whereby the intensity ratio increased as the reaction time of the polymer wrapping increased.

### 3.4. High-Resolution Transmission Electron Microscopy Analysis

**Figure 6:**
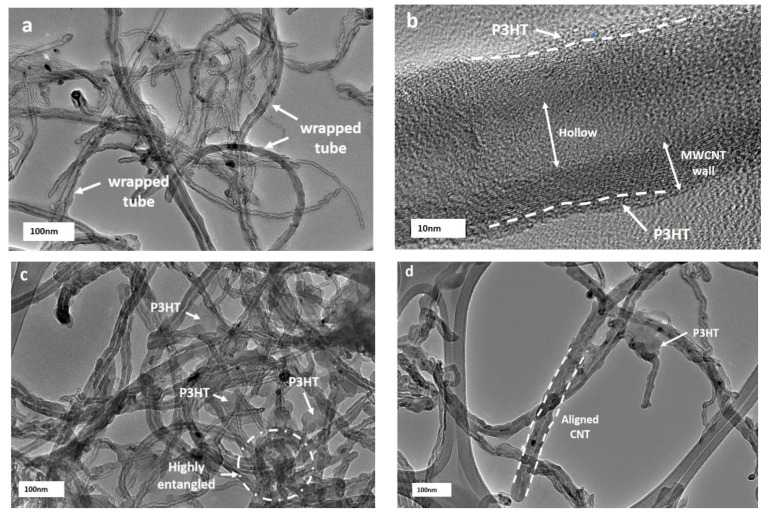

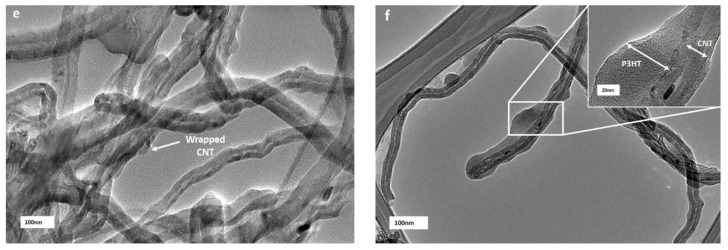
HRTEM image of P3HT/MWCNT-OH nanocomposites at different reaction times (**a**) 24 h, (**b**) 24 h at 10 nm view (**c**) 48 h, (**d**) 72 h, (**e**) 96 h, and (**f**) 120 h.

In order to have a better resolution of the behaviour of individual nanotubes wrapped/unwrapped with P3HT, the nanocomposite was imaged using HRTEM. A close analysis of isolated nanocarbon confirmed that MWCNT-OH dispersed well in the polymer matrix. From the images in Figure 6, at lower reaction times of 24 h and 48 h during the preparation of nanocomposites, it could be seen clearly that there was a heterogeneous distribution of MWCNT-OH in the polymer matrix. There were unwrapped and non-uniformly wrapped P3HTs on nanotubes that adhered to the MWCNT-OH sidewalls with a diameter of about 30 nm. This slightly expanded the diameter of nanotubes, which were close to the diameter of pristine MWCNT-OH, and this might explain that a longer reaction time was required to enhance the interaction between P3HT and MWCNT-OH. Such ununiform P3HT wrapped over the MWCNT-OH sidewall could be attributed to a low electronic interaction between the lone pair of sulphur ions in P3HT and the π-bond from MWCNT-OH [63], whereas nanocomposites that were prepared at 72- and 96-hour reaction times showed that the nanotubes were dispersed homogenously with less entanglement and more aligned tubes. This might be due to sufficient time for the P3HT to swell, make an interaction and wrap the MWCNT-OH sidewalls. The formation of highly uniform and thick coatings of the P3HT layer was found on the sidewall of MWCNT-OH, which accounted for the stability of the P3HT/MWCNT-OH nanocomposites. This might be explained by the non-covalent of π–π interaction and CH-π interaction, which occurred on the wrapping of P3HT over the MWCNT-OH that resulted in uniform dispersion by overcoming the van der Waals forces between the MWCNT-OH [48].

However, the maximum reaction time at 120 h might experience a different behaviour of the nanocomposite texture. The apparent formation of excess P3HT on the surface that resulted in thickening of the diameter of nanotubes with ununiform wrapping occurred at a longer reaction time. This was in agreement with the Raman spectra analysis and FESEM images. From Figure 6, it was observed that at a lower reaction time of 24-hour preparation of nanocomposites, there were unwrapped parts of MCNT-OH by the P3HT and non-uniform wrapping, which adhered to the MWCNT-OH sidewalls with a diameter of 30 nm, which was around the diameter of pristine MWCNT-OH. Such ununiform P3HT that wrapped over the MWCNT-OH sidewall could be accredited to less electronic interaction between the lone pair of sulphur ions in P3HT and the π-bond from MWCNT-OH [63]. This showed that a longer reaction time of the suspension was needed for better interaction between P3HT and MWCNT-OH. A longer reaction time obviously showed a reduction in the parts of unwrapped MCNT-OH. This might be due to sufficient time for the P3HT to swell, form an interaction and then wrap the MWCNT-OH sidewalls. Hussein et al. (2020) suggested that highly efficient π-conjugated systems might facilitate extra movement of charge carriers, as the nanocomposites would show a significant improvement in the direct current (DC) electrical conductivity, electrochemical behaviour, sensing ability, and the limit of detection (LOD) [64].

### 3.5. Thermogravimetric Analysis

**Figure 7:**
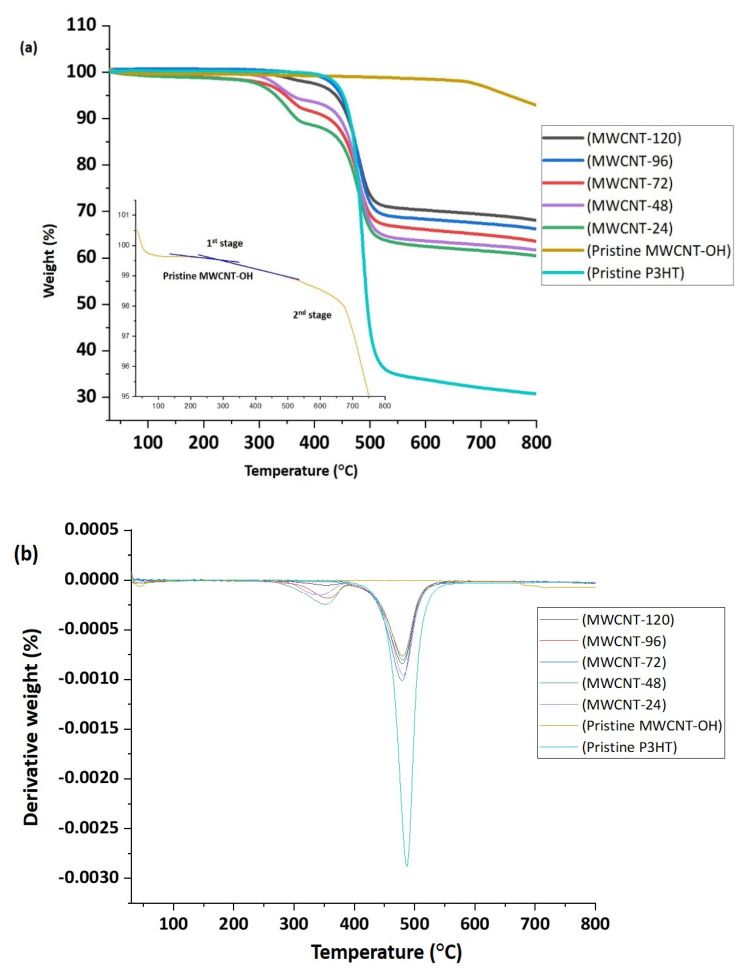
(**a**) TGA (**b**) DTG curves for pristine P3HT, pristine MWCNT-OH, and P3HT/MWCNT-OH nanocomposites at different reaction times.

The thermogravimetric analysis (TGA) and the differential thermogravimetry (DTG) curves for pristine P3HT, pristine MWCNT-OH, and P3HT/MWCNT-OH nanocomposites at different reaction times are presented in Figure 7a,b. A single degradation step was observed for pristine P3HT, which corresponded to the degradation of the alkyl side chain and main chain backbone at a temperature of 487.2 °C accompanied by a symmetric exothermic peak. Hacaloglu et al. (1997) stated that the degradation referred to the unsaturation along the chain, and the breaking of the thiophene ring was assigned to be the most possible decomposition pathway [65]. The MWCNT-OH was comparatively very stable in the range of 0 to 900 °C, with only 1.3% weight loss recorded at a temperature above 600 °C (2nd stage). This corresponded to the degradation temperature of impurities, C=C and C–OH [36]. Peng et al. (2008) indicated that the degradation of MWCNT significantly commenced at a temperature above 599.85 °C [66]. The 1st stage of decomposition of MWCNT-OH was observed at a temperature of 310.5 °C, which corresponded to the removal of hydroxyl groups on MWCNT [67,68]. Overall, based on the T_onset_ (Table 4), the thermal stability of the P3HT/MWCNT-OH was improved when the reaction time increased. This might have been influenced by the sufficient time for the P3HT to swell, form an interaction and then wrap the MWCNT-OH sidewalls that finally led to the improvement of thermal stability. This stage might be due to the chain scission of the sulfur atoms from the P3HT. However, the onset temperature for the P3HT/MWCNT-OH for all samples was observed from 342.7 to 355.5 °C.

It was observed that there was no significant trend in the degradation temperature for the T_max_ from the P3HT/MWCNT-OH nanocomposites. At this stage, the degradation, which occurred for P3HT/MWCNT-OH nanocomposites, might correspond to the side chain and the polymer backbone degradation. The thermal stability of P3HT/MWCNT-OH upon increased reaction time also supported the observation for T_10%_ and T_25%_, which referred to the degradation temperature at 10% of weight loss and 25% of weight loss, respectively. While at a temperature of 500 °C, the weight loss percentage was reduced as the reaction time increased from 24 h to 120 h. Compared to pristine P3HT, a reduction of weight loss was observed, from 55 to 26.1%. In addition, the phenyl ring in the P3HT backbone could increase thermal stability due to its role in the conjugation length. This proved that the effect of reaction time might influence the thermal stability and the weight loss of the nanocomposites. As a result, the greater thermal stability of the nanocomposites could result in the excellent stability of the conductivity of P3HT/MWCNT-OH nanocomposites at higher temperatures. The results in Table 4 show that the thermoelectric performance of the nanocomposites could be investigated under 300 °C, without destroying the structure of P3HT. 

## 4. Conclusions

In this study, non-covalent functionalisation of a P3HT-wrapped MWCNT-OH nanocomposite was successfully prepared. It was found that the textural, structural, morphological, and thermal stability of the resultant nanocomposite properties changed significantly as the reaction time during the stirring process increased from 24 h to 120 h. The HRTEM and FESEM images revealed the incorporation of P3HT wrapped homogenously on the MWCNT wall at a lower reaction time with thickness in the range of 6 to 8 nm, whereas as the reaction time increased, the distribution of P3HT wrapped on the CNT wall became less homogeneous with the distribution of a thick P3HT layer of 20 to 23 nm wrapped onto the outer walls in certain areas. The intensity ratio from the Raman spectra increased up to 19% at a longer reaction time of 120 h compared with pristine MWCNT-OH, which caused the D band to increase and lowered the G band, which provides evidence of successful dispersion and wrapping of MWCNT-OH into the P3HT matrix. The stability of the nanocomposites increased as the reaction time increased, which influenced the thermal stability and the weight loss of the nanocomposites. In addition, this result would be expected for an effective electronic as well as π–π interaction between the P3HT and MWCNT-OH system that prolonged the degradation of the composite system at a certain point. Therefore, this study suggests that greater thermal stability and electronic interaction of the nanocomposites could drive the excellent potential application in the fabrication of polymer-based devices.

## Figures and Tables

**Table 1:** Intensity ratio of the I_D_/I_G_ band of pristine MWCNT-OH and P3HT/MWCNT-OH nanocomposites.

Sample	I_D_	I_G_	I_D_/I_G_
Pristine MWCNT-OH	1297	1571	0.83
MWCNT-24	1312	1458	0.90
MWCNT-48	1313	1414	0.93
MWCNT-72	1372	1456	0.94
MWCNT-96	1390	1431	0.97
MWCNT-120	1398	1427	0.98

**Table 2:** XPS data for pristine P3HT, pristine MWCNT-OH, and P3HT/MWCNT-OH nanocomposites.

Sample	Peak	Binding Energy (eV)	Atomic (%)	Ref.
Pristine P3HT	C 1s	284.6	-	[59]
	O 1s	~530.0	-	
	S 2s	229.1	-	
	S 2p	165.1, 165.8	-	
Pristine MWCNT-OH	C 1s	284.0	97.25	Current work
	O 1s	531.0	2.75	
	S 2s	-	-	
	S 2p	-	-	
MWCNT-72	C 1s	284.0	80.5	
	O 1s	531.0	2.7	
	S 2s	227.0	8.5	
	S 2p	163.0	8.3	
MWCNT-96	C 1s	281.0	78.2	
	O 1s	529.0	2.78	
	S 2s	225.0	6.6	
	S 2p	161.0	12.5	
MWCNT-120	C 1s	281.0	77.07	
	O 1s	529.0	2.63	
	S 2s	225.0	6.5	
	S 2p	161.0	13.8	

**Table 3:** Mean diameter of P3HT/MWCNT-OH nanocomposites at different reaction times.

Sample	Diameter (nm)	Standard Deviation
Pristine MWCNT-OH	27	8.10
MWCNT-24	33	12.48
MWCNT-48	35	14.80
MWCNT-72	42	18.30
MWCNT-96	45	12.58
MWCNT-120	50	19.24

**Table 4:** Characteristic temperatures of nanocomposites at elevated weight loss.

Sample	T_onset_ (°C)	T_max_ (°C)	T_10%_ (°C)	T_25%_ (°C)	Weight Loss (%) at 500 °C
Pristine P3HT	-	487.2	462.9	479.6	55.0
Pristine MWCNT-OH	310.5	-	-	-	1.3
MWCNT-24	342.7	481.0	364.8	478.4	33.8
MWCNT-48	351.3	480.7	446.1	483.5	31.7
MWCNT-72	351.5	481.0	450.2	488.9	31.0
MWCNT-96	354.5	480.2	455.4	490.0	30.5
MWCNT-120	355.5	479.5	461.5	493.8	26.1

## Data Availability

Not applicable.
